# A patient presenting with abdominal pain to the general practitioner: a case report

**DOI:** 10.1186/1757-1626-2-9297

**Published:** 2009-12-09

**Authors:** Thomas Frese, Sven Jonas, Hagen Sandholzer

**Affiliations:** 1 Department of Primary Care of the Leipzig Medical School, Philipp-Rosenthal-Straße 55, 04103 Leipzig, Germany; 2 Division of Visceral-, Transplantation-, Thoracic- and Vascular-Surgery of the Leipzig University Hospital, Liebigstraße 20, 04103 Leipzig, Germany

## Abstract

**Introduction:**

Right-sided upper abdominal pain is a common cause of presentation to general practitioners.

**Case presentation:**

An otherwise well 46-year-old woman presented to her general practitioner with intermittent abdominal pain that had been present for several months. The only abnormality found at the initial consultation was moderate tenderness in the right upper abdomen. The laboratory tests that were ordered showed elevated parameters of inflammation. Sonography suggested the presence of an echinococcal cyst in segment VIII of the liver. Computed tomography confirmed this finding and showed no other cysts. On the basis of serological tests and the clinical findings, a diagnosis of *Echinococcus granulosus *infection was made. The patient was therefore admitted to hospital for surgical removal of the cyst. Her postoperative recovery was without complication and she remained free of symptoms.

**Conclusion:**

*Echinococcus granulosus *infections are rare in Germany, with an incidence of 1:1,000,000. The sonographic appearances are generally characteristic and permit diagnosis. Treatment is pharmacological (albendazole, mebendazole) and surgical. It is curative in the vast majority of cases. The possibility of echinococcal infection should be considered in patients, especially immigrants, with abdominal pain.

## Introduction

Abdominal pain is a common cause of presentation to general practitioners. According to data from Part 2 of the *Sächsische Epidemiologische Studie in der Allgemeinmedizin *(Saxon epidemiological study in general practice; SESAM-2), abdominal pain accounts for 4.2% of visits to general practitioners. The present case report refers to a cause of abdominal pain which, though relatively rare, must be considered by general practitioners in the differential diagnosis of abdominal pain.

## Case presentation

Mrs. S., a 46-year-old woman from Azerbaijan who had lived in Germany for the past eight years, had always been healthy and had never been under the care of a general practitioner. In November she visited a general practitioner complaining of right upper abdominal pain with no radiation. The pain, which was described as pressing in character, had been present for about three months and was intermittent. There was no nausea, vomiting, weight loss, melaena, change in bowel habit, urinary symptoms, or fever. The patient denied alcohol abuse and said that she was a nonsmoker and took no regular medication.

Blood pressure 140/80 mmHg, weight 75 kg, height 170 cm. Heart and lungs normal to percussion and auscultation. No lymph node enlargement. Abdomen slightly obese and soft. Tenderness present in the right upper abdomen. Bowel sounds normal. Renal angles clear. The clinical findings did not indicate a need for immediate hospital admission. On the basis of the history and the findings at the initial consultation, a series of laboratory tests and an ultrasound scan of the abdomen were ordered.

**Figure 1:**
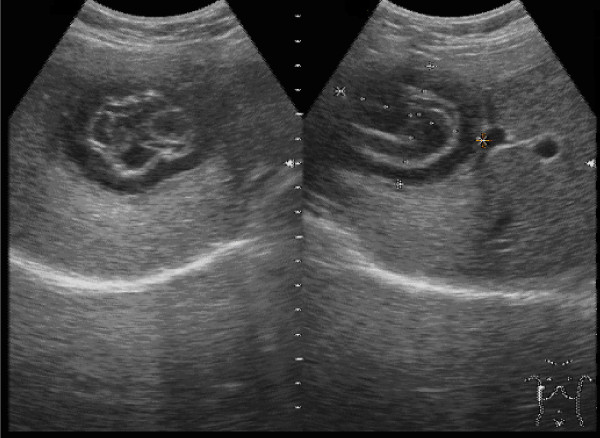
**Sonographic appearance of the echinococcal cyst in hepatic segment VIII**.

**Figure 2:**
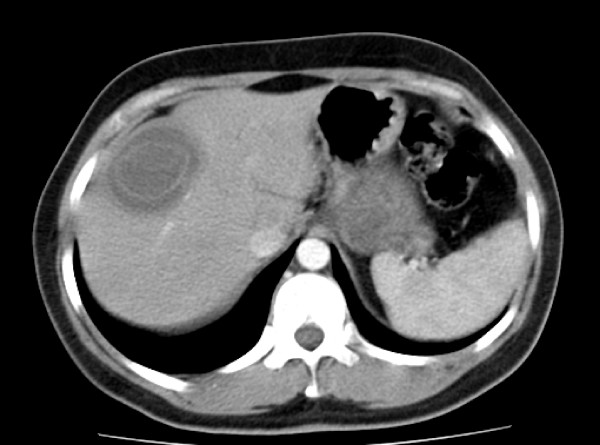
**2 Computed tomographic appearance of the echinococcal cyst in hepatic segment VIII**.

Relevant abnormal laboratory test results: C-reactive protein 108 mg/l (normal range < 5 mg/l), erythrocyte sedimentation rate 91 mm after two hours (normal range < 20 mm). The following values were within normal limits: blood count, aspartate aminotransferase, alanine aminotransferase, gamma-glutamyltransferase, alkaline phosphatase, creatinine, bilirubin, serum electrolytes. Sonography showed an irregularly echogenic, partially calcified septate cystic structure in segment VIII of the liver (Fig. 1). No other abnormalities were apparent. In view of the sonographic findings, the radiologist arranged for the immediate performance of a contrast-enhanced computed tomographic scan of the abdomen. This showed a circumscribed rounded lesion measuring 5.8 × 4.7 × 5 cm in hepatic segment VIII (Fig. 2). The lesion showed a thick rim of calcification and a suggestion of fine septa with no affinity for contrast medium. The liver was otherwise homogeneous and of normal appearance. The other parenchymal abdominal organs appeared normal and there were no enlarged lymph nodes.

Additional laboratory tests showed an antibody titre of 1:512 for *E. granulosus *and the presence of anti-*E. multilocularis *IgG. A western blot analysis was positive for *Echinococcus granulosus *and negative for *Echinococcus multilocularis*. After being informed about possible treatment options, the patient was referred to the university hospital. There she underwent hepatic segmental resection and cholecystectomy. Histological preparation of the material removed at operation showed caseating necrosis with a moderately severe, partly giant-cell, inflammatory reaction at the margin of an echinococcal cyst. There was no evidence of malignancy. The histological appearance was consistent with *Echinococcus granulosus *infection. Moderately severe chronic cholecystitis with moderate fibrosis of the gallbladder wall and cholecystolithiasis were also present. The skin sutures were removed on the tenth postoperative day. As a result of wound dehiscence, the wound healed partly by second intention. The patient was free of symptoms. Follow-up treatment with Eskazole^® ^400 mg (active ingredient: albendazole) b.i.d. was given. The patient's subsequent progress was without complication.

## Discussion

Upper abdominal pain is a common cause of presentation to general practitioners. Provided that the problem is new and that the clinical findings do not indicate a need for immediate hospitalisation, the cause of the pain can be satisfactorily established by history-taking and physical examination, where necessary supplemented by laboratory tests and imaging investigations (sonography) [1]. The SESAM-2 study found the most common diagnoses in patients presenting to general practitioners with abdominal pain to be gastritis and duodenitis (24.5%), while other infectious bowel diseases (7.5%) and cholecystolithiasis and cholecystitis (6.1%) occupied positions four and five in terms of frequency of diagnosis. Echinococcal disease is a rare cause of abdominal pain in Central Europe. In Germany the Robert Koch Institute has received reports of approximately 100 cases of echinococcal disease (60 cases of *Echinococcus granulosus*, 20 of *Echinococcus alveolaris*, and 20 unspecified; oral communication from the Robert Koch Institute) per year over the past few years. The incidence of this notifiable disease is thus approximately 1:1,000,000. The pattern of occurrence of *Echinococcus granulosus *infection in Central Europe is sporadic, whereas in parts of the former Soviet Union it is highly endemic [2].

In the case described here echinococcal infection was diagnosed with the aid of sonography. The distinction between cystic and alveolar echinococcosis is fundamental for treatment and prognosis. Along with imaging investigations, serological testing is essential. Serological screening is based on the use of only slightly purified antigens of *E. granulosus *or *E. multilocularis *and therefore often yields nonspecific results due to cross-reactivity [3]. In the present case the inconclusive serological result was therefore supplemented by western blot analysis for positive identification of the parasite. In conjunction with the computed tomographic findings, this established the diagnosis of a cyst due to *Echinococcus granulosus*. This disease is generally asymptomatic at first. Provided that their diameter remains less than 5 cm, the cysts rarely become symptomatic [4]. The case described here is typical in this respect. The infection gives rise to nonspecific symptoms (abdominal pain, digestive disturbances, nausea, vomiting, weight loss, fever, jaundice) and secondary complications (cyst rupture, anaphylaxis) [4,5]. Asymptomatic calcified echinococcal cysts require no treatment. If symptoms develop, operative treatment is the method of choice [3]. Pre- and postoperative anthelmintic therapy is recommended [4], since it reduces the risk of recurrence and of intraperitoneal dissemination of the infection after rupture or intraoperative damage to the cyst [6]. In the present case albendazole (in the form of Eskazole^®^) was used in accordance with WHO recommendations. A daily dose of 10 to 15 mg of this drug per kilogram bodyweight is taken in two individual doses after meals for a period of up to several months [6].

## Conclusion

In the case described here abdominal pain, a common reason for presentation to general practitioners, was found to be due to a rare condition. Especially in immigrants, the possibility of echinococcosis must be considered in the differential diagnosis of abdominal pain.

## Consent

Written informed consent was obtained from the patient for publication of this case report and accompanying images. A copy of the written consent is available for review by the Editor-in-Chief of this journal.

## Competing interests

The authors declare that they have no competing interests.

## Authors' contributions

TF performed the clinical examination, ordered laboratory investigations, and initiated treatment. SJ performed the surgery and helped prepare the manuscript. HS contributed substantially to the diagnostic investigations and manuscript preparation. All authors read and approved the final manuscript.
